# Tri-modal management of primary small cell carcinoma of the pancreas (SCCP): a rare neuroendocrine carcinoma (NEC)

**DOI:** 10.1186/s12876-021-01901-7

**Published:** 2021-09-03

**Authors:** Safa Elzein, Fei Bao, Ray Lin, Gabriel Schnickel, Andrew M. Lowy, Gregory P. Botta

**Affiliations:** 1 Department of Internal Medicine, Scripps Clinic/Green Hospital, 10666 N. Torrey Pines Road, La Jolla, CA 92037 USA; 2 Department of Pathology, Scripps Clinic/Green Hospital, 10666 N. Torrey Pines Road, La Jolla, CA 92037 USA; 3 Scripps Health Radiation Oncology, 10666 N. Torrey Pines Road, La Jolla, CA 92037 USA; 4 Division of Surgical Oncology, Department of Surgery, Moores Cancer Center, University of California San Diego, 3855 Health Sciences Road, La Jolla, CA 92037 USA; 5 Department of Medicine, Division of Hematology/Oncology, Moores Cancer Center, University of California San Diego, 3855 Health Sciences Road, La Jolla, CA 92037 USA

**Keywords:** Small cell, Carcinoma, Pancreas, Neuroendocrine carcinoma

## Abstract

**Background:**

Primary small cell carcinoma of the pancreas (SCCP) is a rare malignant neuroendocrine carcinoma (NEC). Typically, it presents with lymphovascular invasion as well as metastasis at the time of diagnosis which portends a dismal prognosis. Treatment is typically based on therapy used for other aggressive NECs such as small cell lung cancer. Although multimodal surgery, radiation and chemotherapy may improve prognosis, the outcome generally remains poor.

**Case presentation:**

Here we present a primary SCCP managed with neoadjuvant multi-agent chemotherapy combined with radiotherapy and surgery

**Conclusions:**

Multi-disciplinary therapy resulted in an ongoing 28 + month radiographic complete response and overall survival.

## Background

Based upon the World Health Organization (WHO) 2019 classification, pancreatic neuroendocrine neoplasms (NENs) are broadly classified into neuroendocrine tumors (NET) and neuroendocrine carcinomas (NEC) [1]. The 5-year overall survival (OS) of pancreatic NET all-comers is considerably improved versus pancreatic ductal adenocarcinoma (PDAC) all-comers (47-54% vs 5-9%) ) [2, 3]. Poorly differentiated, high grade neuroendocrine carcinomas (NECs) are distinguished from well differentiated, low grade NETs by a Ki-67 index of > 20% [4]. Moreover, pancreatic NECs are further classified into large-cell, small cell, or combined [5]. The majority of pancreatic NECs are either large cell or combined [6]. Conversely, primary small cell carcinoma of the pancreas (SCCP) is an extremely rare NEC entity. In a recent retrospective analysis of over 30 million records in the National Cancer database from 1998 to 2011, SCCP accounted for only 0.2% of all pancreatic tumors [7]. The biologic origin of SCCP is obscure and was first described in 1972 by Patchefsky et al. as a case of pancreatic “oat” cell tumor with increased levels of urinary 5-hydroxy indoleacetic acid (5-HIAA) suggesting the possibility of pancreatic islet origin [8]. Modern paradigms postulate that pancreatic NECs grow from stem cells found in the ductal system of the pancreas which have the capability of transforming into endocrine cells [9].

## Case presentation

**Fig. 1:**
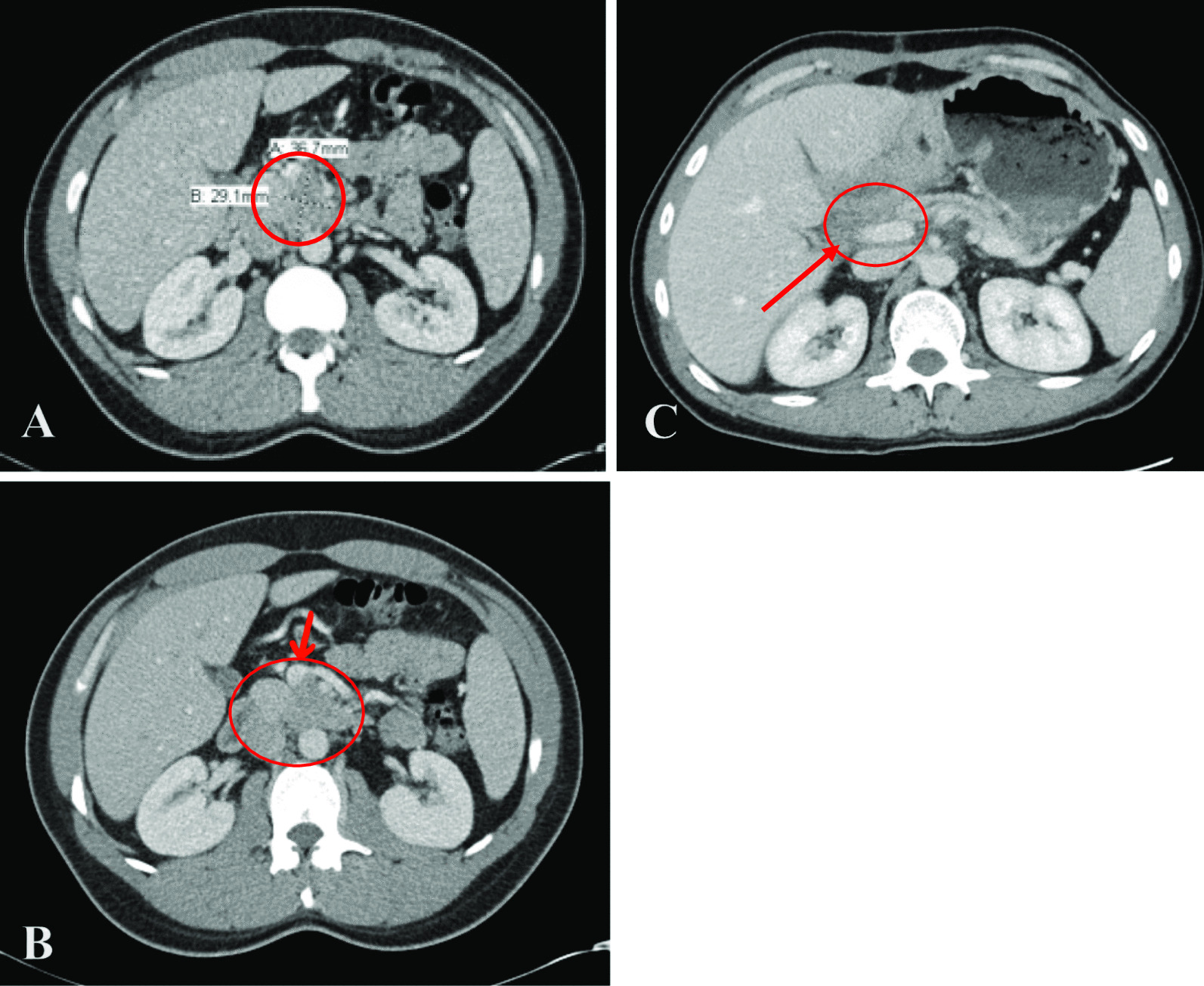
**A** Pre-operative CT scan of abdomen and pelvis revealing a 3.7 × 2.9 cm ill-defined Pancreatic head /uncinate process mass (red circle). **B** Pre-operative CT scan of the abdomen and pelvis observing the superior mesenteric vein (SMV, red arrow) prior to resection of tumor (within red circle). **C** Post-operative CT scan of abdomen and pelvis, showing residual soft tissue (red circle) encasing the superior mesenteric vein (red arrow) but without evidence of definitive disease

**Fig. 2:**
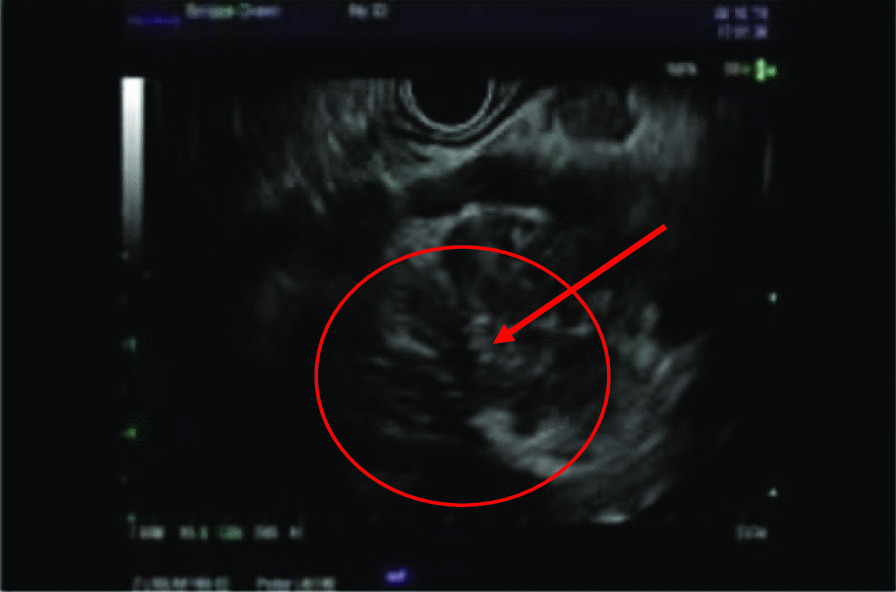
EGD-EUS revealing irregular hypo-echoic mass of the uncinate process (red arrow) of the pancreas (red circle) with involvement of the superior mesenteric vein

A 29-year-old man with no significant past medical history presented to the hospital with epigastric pain radiating to the mid-thoracic vertebrae of one-month duration after an extended car ride. The pain was precipitated after meals, by exercise, and with alcohol intake. The patient reported sporadic alcohol use of 4 drinks per week at baseline, however the weekend prior to this hospital visit he consumed 6 drinks during a wedding. He denied any history of tobacco use or use of other drugs or supplements. The patient complained of nausea without vomiting and mild weight loss in the setting of his cross-fit training. He described no changes to stool or urine consistency or color and further denied changes in skin tone or sclera color. The patient noted an increase in anxiety-like symptoms (tachycardia and tachypnea) for approximately 4 months prior to his presentation. Laboratory studies were within normal limits apart from an elevated lipase of 1434 units/L. The admitting internal medicine team initially managed his case as acute pancreatitis with IV fluids, pain control and NPO (nil per os) status. Computerized tomography (CT) of the abdomen disclosed an ill-defined 3.7 × 2.9 cm mass in the head of pancreas (Fig. 1A). The gastrointestinal (GI) team was consulted for an upper esophagogastroduodenoscopy with endoscopic ultrasound (EGD-EUS) which revealed an irregular hypo-echoic mass of the uncinate process of the pancreas with involvement of the superior mesenteric vein (SMV) (Fig. 2). Multiple intra-abdominal lymph nodes in the peri-gastric and porta hepatis region were observed. A transgastric biopsy of the peri-gastric lymph node was obtained rather than from the pancreatic mass as it was more easily accessible.

**Fig. 3:**
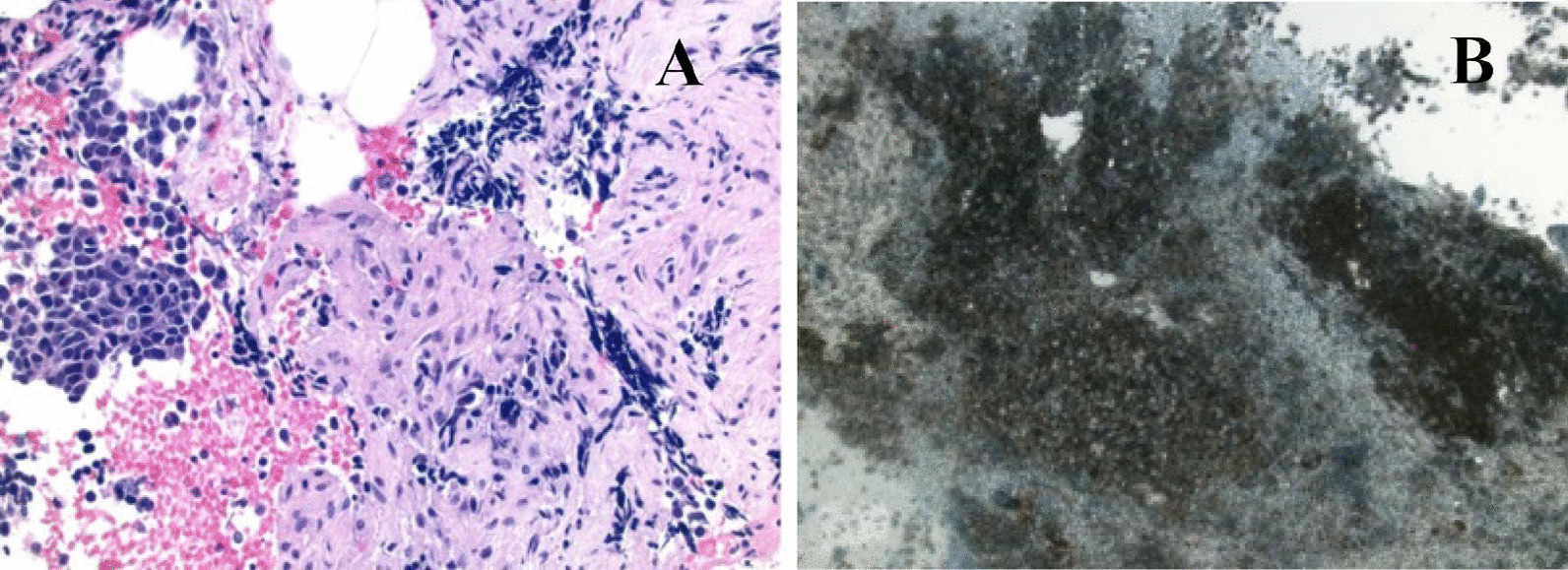
**A** Lymph node biopsy showing high grade neoplasm composed of neoplastic cells with salt and pepper nuclear chromatic and high nuclear to cytoplasmic ratio with focal crush artifact. **B** Ki-67 proliferative index of > 80% by manual quantitation

**Fig. 4:**
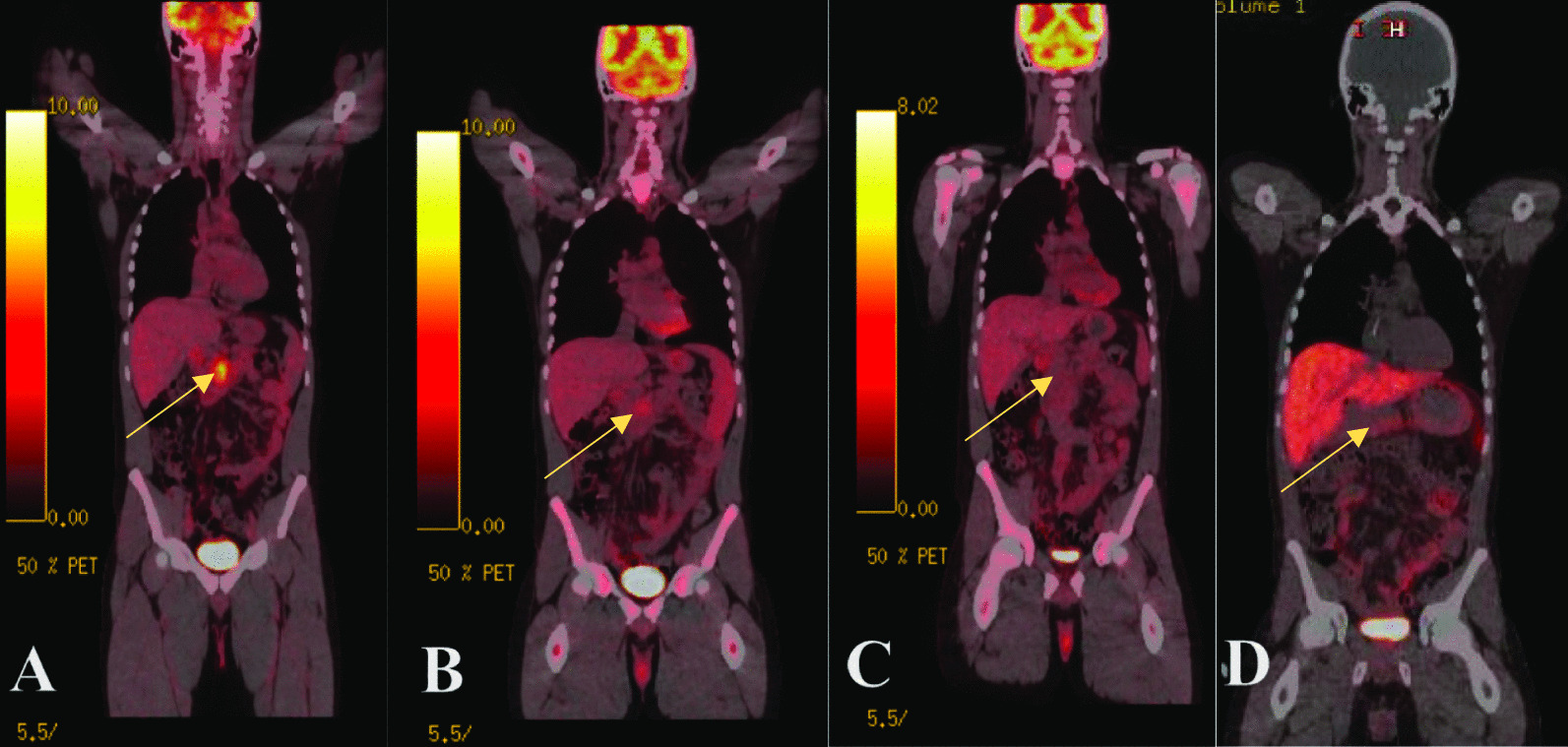
**A** PET scan prior to treatment (4/23/2019) showing moderate FDG avidity in the pancreatic mass, with an SUV max reaching 7.5 g/mL (Arrow). There is some uptake in adjacent nodes. **B** PET scan after 3 cycles (6/6/2019) of cisplatin/etoposide showing decrease in the PET activity involving the pancreatic mass with a maximum SUV value of 3.2 versus 7.6 on prior examination (Arrow). The adjacent peripancreatic PET avid lymph node also demonstrates decreased activity. **C** PET scan after 6 cycles (9/20/2019) of cisplatin/etoposide showing resolution of mild residual uptake in the peripancreatic foci (Arrow). **D** Post-operative PET Gallium without definite increased radiotracer uptake in the surgical bed to suggest DOTATATE-avid residual disease. Low level activity along the pancreatic remnant is nonspecific

Pathologic analysis of the biopsy revealed a high grade, poorly differentiated neoplasm (Fig. 3A) and immunohistochemical studies revealed tumor cells positive for AE1/AE3 and synaptophysin while negative for CD3, CD20, PAX5, chromogranin, TTF-1, CDX2 and CDH17. Ki-67 analysis revealed a proliferative index greater than 80% by manual quantitation consistent with a final diagnosis of small cell carcinoma of the pancreas (SCCP) (Fig. 3B). Tissue next generation sequencing (NGS) and immunostaining was unable to be completed at diagnosis as the tissue sample size was insufficient for analysis. Blood NGS (Tempus, https://www.tempus.com) at diagnosis found no evidence of circulating actionable mutations(Table 1). Staging CT scan of the chest did not reveal evidence of a primary lung tumor or metastases to the lungs. Brain Magnetic Resonance Imaging (MRI) was obtained at diagnosis due to clinical symptoms of ongoing headaches and was negative for evidence of metastatic disease within the central nervous system. Additional laboratory studies including IgG subclass 4, Carbohydrate antigen 19-9 (CA19-9), Carcinoembryonic antigen (CEA), chromogranin A (CgA) and neuron specific enolase (NSE) were within normal limits (Table 2). An initial pre-treatment Positron Emission Tomography–Computed Tomography (PET/CT) scan demonstrated moderate fluorodeoxyglucose (FDG) avidity in the pancreatic mass with some uptake in adjacent nodes but no evidence of FDG avid metastases (Fig. 4A).

**Table 1:** Tumor characteristics

Testing platform	Specimen	Specimen collection date	Findings
Tempus	Blood	Pre-operative	None
Paradigm	Tissue	Post-operative	MGMT 3 + PDL1:TILs 0% PDL1:Tumor 0% PTEN 4 + TRKpan 2 +
Guardant	Blood	Post-operative	None
Foundation	Tissue	Post-operative	TP53 Y236C
Invitae	Blood	Post-operative	No Germline Mutations
Natera	Tissue and Blood	Post-operative	ctDNA negative

**Table 2:** Tumor markers

Tumor marker	Value	Normal range
CA19-9	10 U/mL	30–42 U/mL
CEA	1.4 ng/mL	< 3.8 ng/mL
Chromogranin A	72 ng/mL	0–103 ng/mL
Neuron specific enolase	5.9 µg/L	3.7–8.9 µg/L
IgG4	16 mg/dL	1–123 mg/dL

Due to the significant local tumor burden, aggressive biology, and patient age, the case was presented to a multidisciplinary GI tumor board where it was decided to initiate neoadjuvant systemic chemotherapy after which, in the setting of disease control, an attempt at surgical resection would be considered. The patient had an initial Eastern Cooperative Oncology Grade. (ECOG) of 1 due to symptoms of his tumor and received 3 cycles of cisplatin/etoposide followed by an interval PET scan which observed a reduction in PET avid FDG (Fig. 4B). The patient was again discussed at a multi-disciplinary GI tumor board and due to radiologic evidence of ongoing soft tissue surrounding the superior mesenteric artery on imaging, a course of radiotherapy to a dose of 5040 centi-gray (cGY) was recommended prior to any attempted resection. The patient completed the prescribed radiotherapy concurrently with his fourth cycle of Cisplatin/Etoposide and a follow up CT scan showed stable disease at the uncinate process: the 1.3 cm hypoenhancing mass with abutment of the superior mesenteric artery and vein by less than 180 degrees. Chemotherapeutic tolerance was reduced by cycles 5 and 6 secondary to fatigue, cachexia, and nausea, therefore his ECOG was graded as a 2. After cycle 6, a final PET/CT showed resolution of the peripancreatic FDG avidity. No intra or extrahepatic bile duct dilation was seen and the uncinate process mass was slightly smaller than previous assessments. There was abutment of the superior mesenteric artery and vein by less than 180 degrees and no celiac truck or main portal vein involvement. No pancreatic duct dilation was evident. (Fig. 4C). The decision was made to proceed to an attempt at surgical resection after 6 weeks of chemoradiation ‘washout’ permitting a performance status return to ECOG 1.

The patient underwent a pancreaticoduodenectomy (‘Whipple surgery’) five months following initiation of chemotherapy and radiotherapy. Intraoperatively, exploration of the abdomen revealed no metastatic disease to the liver or peritoneal surface. The tumor was palpable in the inferior portion of the pancreatic head and uncinate process while the pancreatic parenchyma was soft with pancreatic duct dilation to 2–3 mm. The tumor involved the pancreatic head and uncinate process as well as the superior mesenteric vein (SMV) with abutment of the superior mesenteric artery. The NEC was removed from the artery with limited difficulty but was adherent to the vein and required detailed separation. The tumor was adherent to the SMV at the level of the first jejunal branch. The jejunal branch was ligated and upon removing the specimen a venotomy was created in the SMV. This was repaired primarily with 5-0 prolene. The Whipple reconstruction was then completed. At that time the bowel was markedly edematous concerning for outflow obstruction. The gastrojejunostomy, hepaticojejunostomy and pancreaticojejunostomy were taken down to further evaluate the SMV. The vein was felt to be obstructed due to narrowing at the repair and progressing thrombosis. The SMV was then clamped and reconstructed with a 1 cm × 4 cm bovine pericardial patch after Fogarty thrombectomy. Following reconstruction the vein was interrogated by doppler which noted excellent flow and the bowel edema subsided. The Whipple reconstruction was then completed again. His post-operative course was complicated by a bacteremia of unclear etiology necessitating 4 weeks of intravenous antibiotics, but he otherwise recovered uneventfully.

The final pathologic diagnosis post-resection showed a high grade, poorly differentiated (G3) neuroendocrine carcinoma with extensive perineural invasion and lymphovascular invasion. The NEC was diffusely infiltrative and extended into peripancreatic soft tissue with a size of 3.2 × 2.4 × 2.2 cm. There was an uncinate margin microscopically positive for carcinoma. Four of 26 lymph nodes were positive for metastatic carcinoma (4/26) with a final pathological staging: ypT2 N2. There was residual cancer with evident tumor regression, but more than single cells or rare small groups of cancer cells (partial response, score 2). The tumor cells were positive for synaptophysin and negative for chromogranin with a post-treatment Ki-67 proliferation index of 5%.

Post-operative NGS on the resected tumor from two separate companies (Paradigm, https://www.paradigmdx.com and Foundation Medicine, https://www.foundationmedicine.com) revealed no actionable mutations (TP53 Y235C only) and notably the programmed death ligand-1 (PD-L1) expression was negative. Post-operative blood NGS (Guardant, https://guardanthealth.com) found no specific circulating mutations and germline testing (Invitae, https://www.invitae.com) found no heritable mutations. The patient had whole exome sequencing (WES) of the resected tumor performed along with immunostaining and there was no evidence of a personalized circulating tumor DNA (ctDNA) signature detected within his blood 12 months after surgical resection (Natera, https://www.natera.com) (Table 1).

Although the patient’s insurance denied a post-operative FDG-PET, the post resection pathology showed a low proliferative rate. As such, a PET-Gallium scan was completed showing no residual disease uptake (Fig. 4D). DOTATATE PET/CT is typically more sensitive for tumors with Ki-67 index < 30% while FDG PET/CT may demonstrate greater sensitivity for high grade, poorly differentiated malignancy. A post-operative CT scan of abdomen and pelvis at one week postoperatively showed residual soft tissue encasing the superior mesenteric vein (SMV) but without evidence of definitive disease (Fig. 1B). As pathology showed a positive margin, there was a concern for residual tumor. He was referred to radiation oncology to discuss adjuvant radiotherapy for the positive uncinate margin. It was determined that there was no clear target for radiation and his prior neoadjuvant chemoradiation would increase side effects with further courses of radiation. It was discussed that if additional follow up imaging revealed recurrent disease, additional radiation could be reconsidered. Repeat CT imaging at 3 and 6 months post-resection has continued to demonstrate stable soft tissue at the SMV without definitive evidence of recurrence. He currently remains radiographically tumor free, blood NGS mutation free, and ctDNA free now 28 + months following diagnosis after completing tri-modal chemotherapy, radiation, and surgery.

## Discussion and conclusions

Patients with SCCP usually present with clinically diagnosed lymph node and liver metastasis along with pathologically diagnosed perineural invasion [6]. The diagnosis of SCCP is based on histologic analysis of a tumor biopsy with markers of neuroendocrine differentiation (synaptophysin and/or chromogranin A, as well as CD56 expression) and determination of mitotic index (Ki-67). Poorly differentiated, high grade pancreatic NECs are highly proliferative and tend to metastasize prior to clinical presentation, and thus have a poor prognosis. Patient workup consists of imaging for distant metastases using FDG PET (due to an elevated Ki-67) and evaluation of common neuroendocrine tumor markers such as chromogranin A (CgA) and neuron specific enolase (NSE). CgA can be a marker in the diagnosis of gastrointestinal pancreatic neuroendocrine tumors (NETs), but its utility can be confounded by ingestion of foodstuff, proton pump inhibitors, anti-histamines, and selective serotonin reuptake inhibitors [10, 11]. The expression of secretory vesicles in neuroendocrine cells determines chromogranin expression however, and in a de-differentiated NEC, the utility of this marker in evaluating treatment efficacy is questionable [12].

NSE is less specific but was previously found to be associated with survival in NEC [13]. Abnormal CA 19-9 levels are more common in pancreatic ductal adenocarcinomas (PDACs) than in pancreatic NECs and may have the potential for differentiating pancreatic NEC from PDAC. While pancreatic NETs have a low Ki-67 proliferative index and are readily imaged by PET Gallium DOTATATE scans, NECs have a high Ki-67 proliferative index and are more readily imaged by FDG PET. Notably, compared to small cell lung cancer, SCCP does not readily metastasize to the brain. Thus, prophylactic cranial radiation is not the standard of care. In our case, brain MRI obtained on admission due to headaches was negative.

Optimal management of SCCP is based on the established role of cisplatin and etoposide in metastatic small cell lung cancer (SCLC) [9]. Due to the rarity of SCCP, a pulmonary pancreatic metastasis should be ruled out first. In this instance, CT chest did not reveal a primary lung focus. The standard of care is, as always, directed by staging. If local disease alone is present, a common recommendation has been upfront surgical resection followed by 6 cycles of adjuvant cisplatin and etoposide similar to SCLC treatment modalities. We note that there is no prospective data for this recommendation, only retrospective reports stating that cure can be obtained by surgery alone in a minority of localized NEC patients [14, 15]. Further, some patients may have improved overall survival based on palliative cytoreduction even with the eventual development of metastases [16]. If locally advanced or metastatic disease is present, it is recommended to start with systemic chemotherapy prior to a possible resection with cisplatin and etoposide for 4–6 cycles. Some recommend substituting carboplatin and irinotecan [17]. Based on our experience, and the high relapse rate for patients who undergo surgical resection, we recommend a neoadjuvant approach for those with local disease and continuing treatment to maximum response and patient tolerability with an FDG PET scan before treatment, after cycle 3 and after cycle 6. Patients with local disease control and/or complete remissions by PET should be considered candidates for subsequent resection. Those patients with a response but whose local disease is unresectable can initiate active surveillance with serial imaging done on an every 2–3 month basis. If a recurrence is documented after 3 to 6 months of initial treatment, we attempt retreatment with cisplatin and etoposide given the initial sensitivity and long delay between recurrence. Second line regimens include FOLFIRI or FOLFOX while a Temozolomide ± capecitabine trial is currently ongoing (ECOG-ACRIN EA2142) [17, 18]. Topotecan, a common small cell lung cancer drug in the second line setting had previously shown poor results in NEC patients [14].

The IMPower133 study showed improved overall survival with the addition of immunotherapy (atezolizumab) to cisplatin and etoposide in the advanced SCLC setting. These patients additionally continued with maintenance immunotherapy after 6 cycles of chemotherapy regardless of PD-L1 status [15]. This is typically our recommendation for metastatic or unresectable NEC regardless of the tissue of origin as patients with unresectable NEC generally recur after an initial chemotherapeutic response. Ugwu et al. demonstrated the first case of immunotherapy benefit in a patient with SCCP who progressed on platinum-based chemotherapy [16]. Given the rarity of pancreatic NECs, the role of molecular profiling to identify therapeutic targets is being assessed. Vijayvergia et al. noted a high incidence of clinically significant mutations in pancreatic NECs including PIK3CA/PTEN and BRAF [19]. It is unclear if this will result in clinical benefit and requires further research. In our patient, NGS did not reveal a targetable mutation pre- or post-operatively of benefit (Table 2).

Our patient received neoadjuvant chemotherapy combined with radiotherapy followed by surgery and remains stable with 28 + months of overall survival (OS) after diagnosis of SCCP. Survival remains limited in the majority of cases with a reported median OS of 11 months [6]. On the other hand, Winter et al. reported a longer survival in 6 cases of SCCP managed first by surgery and then adjuvant chemo-radiotherapy. In these cases, the median OS was 20 months. Remarkably, one patient lived 173 months representing the longest reported survival for SCCP [20]. Based on these results, we agree that tri-modality therapy consisting of chemotherapy, radiation, and surgery, should be considered in patients with resectable small cell carcinoma of the pancreas. In conclusion, SCCP is a rare pancreatic NEC variant and associated with a poor prognosis. Our patient’s case further supports that surgery after neoadjuvant chemotherapy and radiotherapy may improve prognosis and overall survival in appropriate candidates.

## Data Availability

Patient data was curated from the electronic medical records. The Profile Related Evidence Determining Individualized Cancer Therapy (PREDICT, NCT02478931) database of eligible patients at the Center for Personalized Cancer Therapy (University of California San Diego Moores Cancer Center), whose tissue DNA was analyzed by next-generation sequencing (NGS) was utilized. The datasets analyzed during the current study are available from the corresponding author on reasonable request.

## Abbreviations

5-HIAA: 5-Hydroxyindoleacetic acid
CA19-9: Carbohydrate antigen 19-9
CEA: Carcinoembryonic antigen
CgA: Chromogranin
cGY: Centi-gray
ECOG: Eastern Cooperative Oncology Grade
EGD-EUS: Esophagogastroduodenoscopy with endoscopic ultrasound
FDG: Fluorodeoxyglucose
FOLFIRI: Folinic acid, fluorouracil, irinotecan
FOLFOX: Folinic acid, fluorouracil, oxaliplatin
GI: Gastrointestinal
NEC: Neuroendocrine carcinoma
NET: Neuroendocrine tumor
NGS: Next generation sequencing
NPO: Nil Per Os
NSE: Neuron specific enolase
PDAC: Pancreatic ductal adenocarcinoma
PET/CT: Positron emission tomography–computed tomography
SCCP: Small cell carcinoma of the pancreas
SCLC: Small cell lung cancer
SMV: Superior mesenteric vein
WHO: World Health Organization
